# High-titer rheumatoid factor seropositivity predicts mediastinal lymphadenopathy and mortality in rheumatoid arthritis-related interstitial lung disease

**DOI:** 10.1038/s41598-021-02066-9

**Published:** 2021-11-24

**Authors:** Albina Tyker, Iazsmin Bauer Ventura, Cathryn T. Lee, Rachel Strykowski, Nicole Garcia, Robert Guzy, Renea Jablonski, Rekha Vij, Mary E. Strek, Jonathan H. Chung, Ayodeji Adegunsoye

**Affiliations:** 1 Department of Internal Medicine, University of Chicago, Chicago, IL 60637 USA; 2 Rheumatology, University of Chicago, Chicago, IL USA; 3 Pulmonary/Critical Care, University of Chicago, Chicago, IL USA; 4 Radiology, University of Chicago, Chicago, IL USA

**Keywords:** Rheumatoid arthritis, Respiratory tract diseases

## Abstract

Rheumatoid arthritis-related interstitial lung disease (RA-ILD) is a common connective tissue disease-related ILD (CTD-ILD) associated with high morbidity and mortality. Although rheumatoid factor (RF) seropositivity is a risk factor for developing RA-ILD, the relationship between RF seropositivity, mediastinal lymph node (MLN) features, and disease progression is unknown. We aimed to determine if high-titer RF seropositivity predicted MLN features, lung function impairment, and mortality in RA-ILD. In this retrospective cohort study, we identified patients in the University of Chicago ILD registry with RA-ILD. We compared demographic characteristics, serologic data, MLN size, count and location, and pulmonary function over 36 months among patients who had high-titer RF seropositivity (≥ 60 IU/ml) and those who did not. Survival analysis was performed using Cox regression modeling. Amongst 294 patients with CTD-ILD, available chest computed tomography (CT) imaging and serologic data, we identified 70 patients with RA-ILD. Compared to RA-ILD patients with low-titer RF, RA-ILD patients with high-titer RF had lower baseline forced vital capacity (71% vs. 63%; *P* = 0.045), elevated anti-cyclic citrullinated peptide titer (122 vs. 201; *P* = 0.001), CT honeycombing (50% vs. 80%; *P* = 0.008), and higher number of MLN ≥ 10 mm (36% vs. 76%; *P* = 0.005). Lung function decline over 36 months did not differ between groups. Primary outcomes of death or lung transplant occurred more frequently in the high-titer RF group (HR 2.8; 95% CI 1.1–6.8; *P* = 0.028). High-titer RF seropositivity was associated with MLN enlargement, CT honeycombing, and decreased transplant-free survival. RF titer may be a useful prognostic marker for stratifying patients by pulmonary disease activity and mortality risk.

## Introduction

Rheumatoid arthritis (RA) is the most common systemic rheumatologic disorder, affecting one percent of the United States population^1^. It is characterized by a symmetric polyarthritis of small joints. However patients with RA frequently present with extra-articular disease involving multiple organ systems^2^. Rheumatoid arthritis-related interstitial lung disease (RA-ILD) is the most common extra-articular manifestation of RA, with some studies reporting an incidence as high as 60%^3^. Disease is often progressive, leading to impaired lung function and decreased quality of life^4^. Diagnosis and disease monitoring relies on high-resolution chest computed tomography (CT) imaging. While lung parenchymal appearance may be variable, it is most often characterized by a usual interstitial pneumonia (UIP) pattern^5^. Presence of UIP portends a poor prognosis approaching that of idiopathic pulmonary fibrosis (IPF)^6^. RA-ILD has been recognized as an important driver of morbidity and mortality in the RA population. While overall mortality for RA has decreased over time, RA-ILD mortality has increased significantly in the last several decades and is estimated to be three times higher than in those with RA alone^7–9^. Nonetheless, standardized screening for interstitial lung disease (ILD) among RA patients is not routinely recommended in current guidelines^10^.

There is strong evidence that older age, smoking history, male sex, elevated anti-citrullinated protein (CCP) and rheumatoid factor (RF) titers are known risk factors for developing RA-ILD though the disease mechanism has not been well characterized. Previous studies suggest that RA-ILD pathogenesis involves complex interactions between genetic and environmental factors. Recent data indicates that the immune response against citrullinated proteins in the joints may subsequently lead to pulmonary cross-reactivity^10^. In contrast, others have suggested that autoimmunity in the lung predates autoimmunity at other sites^3^. Current evidence shows that oxidative stress from cigarette smoking, and other environmental exposures further contribute to alveolar injury resulting in the release of cytokines, autoimmune mediators, and activation of pro-fibrotic pathways^11^.

Prognostic factors associated with RA-ILD progression and mortality also remain poorly understood^12^. High concentrations of circulating serum RF and anti-CCP in has been found in patients with RA-ILD, supporting the critical role of these antibodies in disease pathogenesis^13^. Despite this, RF titer is not routinely followed in clinical practice and has not been evaluated for its utility as a prognostic marker in this cohort. Furthermore, mediastinal lymph node (MLN) enlargement, which is seen in up to 70% of patients with RA-ILD, has also previously been associated with decreased transplant-free survival in ILD^14^. We hypothesized that there may be a correlation between the degree of RF seropositivity and RA-ILD progression or mortality, and that patients with the highest RF titers would have MLN changes, and carry the greatest risk of disease progression and mortality in RA-ILD. To determine this, we assessed whether high-titer RF was associated with CT predictors of ILD progression and mortality amongst patients with RA-ILD, independent of lung disease severity at baseline evaluation.

## Materials and methods

### Study design and population

We identified subjects with a diagnosis of connective tissue disease-related interstitial lung disease (CTD-ILD) who had baseline pulmonary function tests (PFT), imaging, and clinical data who were prospectively enrolled in the University of Chicago ILD Registry, and receiving care between 2006 and 2019. Among those we selected eligible individuals with a specific diagnosis of RA-ILD who had a baseline CT chest exam, pulmonary function tests (PFT), and autoimmune serologies collected within one year of diagnosis. Written informed consent was provided by study participants and approval for data collection was granted by the University of Chicago Institutional Review Board (IRB#14,163-A; IRB#16–1062) and all methods were performed in accordance with the relevant guidelines and regulations.

Diagnosis of RA was established by a rheumatologist using ACR/EULAR classification criteria^15^. Diagnosis of RA-ILD and other ILD subtypes was established by multidisciplinary discussion (MDD) among pulmonologists, rheumatologists, chest radiologists and a thoracic pathologist at our institution based on American Thoracic Society/European Respiratory Society criteria for chronic ILD^16^. Subjects without chest CT imaging at diagnosis, baseline PFTs, or serologic data within one year of diagnosis were excluded.

### Data collection

The electronic medical record was retrospectively reviewed to obtain demographic data including age, race/ethnicity, sex, tobacco use, body mass index (BMI), and presence of gastroesophageal reflux in the study population. Anti-nuclear antibody (ANA), anti-CCP IgG, and RF serologies obtained at the time of diagnosis were collected. The time of diagnosis refers to the time at which the multidisciplinary diagnosis of rheumatoid-arthritis associated ILD (RA-ILD) was made following baseline evaluation. PFT data including percent predicted forced vital capacity (FVC), percent predicted forced expiratory volume in 1 s (FEV_1_), FEV_1_/FVC, and diffusing capacity of lung for carbon monoxide (DL_CO_) were obtained at baseline ILD evaluation and in serial epochs of ninety-day intervals over 36 months to assess lung function variation over time for the RA-ILD cohort.

### Radiologic analysis

Our study utilized high-resolution chest CT images (≤ 1.0 mm in thickness in the axial plane, reconstructed with a high frequency algorithm) obtained at our institution at index ILD clinic visit to evaluate for honeycombing, emphysema, and lymphadenopathy. MLNs at stations 1–9 based on International Association for the Study of Lung Cancer criteria were analyzed for size, location, and number^17^. Presence or absence of emphysema and honeycombing as documented on the index chest CT imaging report was used. Although CT honeycombing is the characteristic hallmark of the radiologic usual interstitial pneumonia (UIP) pattern, we limited our analyses to the official report of honeycombing when present on CT scans, for consistency across all subjects. Chest CT interpretation and MLN measurements were performed by two dedicated chest radiologists at the University of Chicago with expertise in ILD. Radiologists were blinded to patient demographic and clinical data. MLN enlargement was defined as a lymph node with a short-axis diameter ≥ 10 mm^18^.

### Data analysis

Comparison of baseline characteristics was performed among patients with RA-ILD and those with other diagnosis of CTD-ILD based on MDD. Patients with RA-ILD were stratified into high-titer and low-titer subgroups based on RF seropositivity. High-titer subgroup was defined as RF ≥ 60 IU/ml^19,20^. Low-titer subgroup was defined as RF < 60 IU/ml. Relevant seropositivity for autoimmune serum markers was defined as ANA titer ≥ 1:320, CCP titer > 3, and RF titer > 14^21^. In sensitivity analyses, patients with RA-ILD were also stratified into high-titer and low-titer subgroups based on CCP titer. High-titer subgroup was defined as anti-CCP ≥ 100 units^19^. High titer anti-CCP and RF thresholds utilized as cut-off points for this study were based on the cited literature, and supported by the clinical experiences of our rheumatologists. Demographic and clinical characteristic comparisons were performed between groups using two-sided t-tests or chi-square tests as appropriate. Mixed regression models were used to assess longitudinal changes over 36 months for serial PFT indices such as FVC, FEV_1_, FEV_1_/FVC, and DL_CO_ measurements. Cox proportional hazard models with log-rank test were used for assessment of transplant-free survival and adjusted for the gender, age and physiology (GAP) score^22^. Statistical significance was defined by *P* value < 0.05. Data analysis was performed using STATA/SE 16.1 for Windows (StataCorp; 2019).

## Results

### Patient characteristics

Of 294 patients with CTD-ILD, we identified 70 patients (24%) with a MDD diagnosis of RA-ILD (E-Table 1). Patients with RA-ILD were more likely to be older (63 ± 10 years vs 57 ± 14 years; *P* < 0.001), have a higher prevalence of tobacco use (64% vs 43%; *P* = 0.003), and report greater pack-year smoking history (23 years vs 8 years; *P* < 0.001) than those with other CTD-ILDs. There was a higher percentage of males among the RA-ILD cohort, however this was not statistically significant (39% vs 27%; *P* = 0.06). Race and baseline pulmonary function did not differ between groups (E-Table 1).

### High-titer RF in RA-ILD

Sixty percent of patients with RA-ILD (n = 42) had high-titer RF seropositivity (RF ≥ 60 IU/ml). There was no significant difference in age, gender, race, BMI, tobacco use, smoking pack-years, or presence of gastro-esophageal reflux among the high-titer and low-titer RF groups (Table 1). Similarly, there were no differences in immunomodulatory therapies administered between the high-titer and low-titer RF groups. Patients in the high-titer RF group demonstrated lower FVC (63.2 ± 15.8 vs 70.8 ± 12.7; *P* = 0.045) but no significant difference in other baseline PFT measures (Table 1). Patients in the high-titer RF group also had significantly higher mean anti-CCP titers (203 ± 99 units vs 113 ± 115 units; *P* = 0.001) but no difference in ANA seropositivity (Table 1).

**Table 1:** Baseline characteristics in RA-ILD.

Characteristics of RA-ILD cohort (n = 70)*	High-Titer RF Present (n = 42)	High-Titer RF Absent (n = 28)	*P*-value
Age, mean (± SD)	63.3 (10.2)	62.1 (9.5)	0.63
Male gender, n (%)	18 (43)	9 (32)	0.81
**Race/Ethnicity**			
Caucasian, n (%)	21 (50)	19 (68)	0.14
African American, n (%)	14 (33)	7.0 (25)	0.66
Tobacco use, n (%)	26 (62)	19 (68)	0.61
Smoking, pk-yrs, mean (± SD)	24.6 (25.3)	20.9 (28.8)	0.58
Gastroesophageal reflux, n (%)	16 (40)	17 (61)	0.09
**Immunomodulatory therapy**			
Prednisone, n (%)	38 (90.5)	23 (82.1)	0.31
Mycophenolate mofetil, n (%)	9 (21.4)	9 (32.1)	0.32
Azathioprine, n (%)	16 (38.1)	13 (46.4)	0.49
Methotrexate, n (%)	27 (64.3)	17 (60.7)	0.76
Other biologic or DMARD therapy^+^, n (%)	36 (85.7)	25 (89.3)	0.66
FVC (% predicted) (± SD)	63.2 (15.8)	70.8 (12.7)	0.045
FEV1 (% predicted) (± SD)	71.2 (19.2)	76.5 (16.7)	0.25
FEV1/FVC (%) (± SD)	77.5 (21.0)	79.1 (12.8)	0.74
DL_CO_ (% predicted) (± SD)	48.9 (20.8)	56.4 (21.6)	0.18
ANA seropositivity, n (%)	20 (54)	14 (52)	0.86
IgG anti-CCP titer (units), mean (± SD)	202.5 (99.4)	112.7 (114.6)	0.001

*Exception: BMI (n = 66); Tobacco use (n = 67); ^+^Other biologic or disease-modifying antirheumatic drug therapy: infliximab, etanercept, adalimumab, hydroxychloroquine, leflunomide, rituximab; FVC, FEV1 (n = 66); FEV1/FVC (n = 65); DLCO (n = 61); ANA = antinuclear antibody seropositivity(n = 64); ANA seropositivity = Titer ≥ 320; CCP = anti-cyclic citrullinated peptide(n = 63); RF = Rheumatoid factor (RF); High-Titer RF Present ≥ 60 IU/ml.

### Chest CT features in RA-ILD

**Figure 1:**
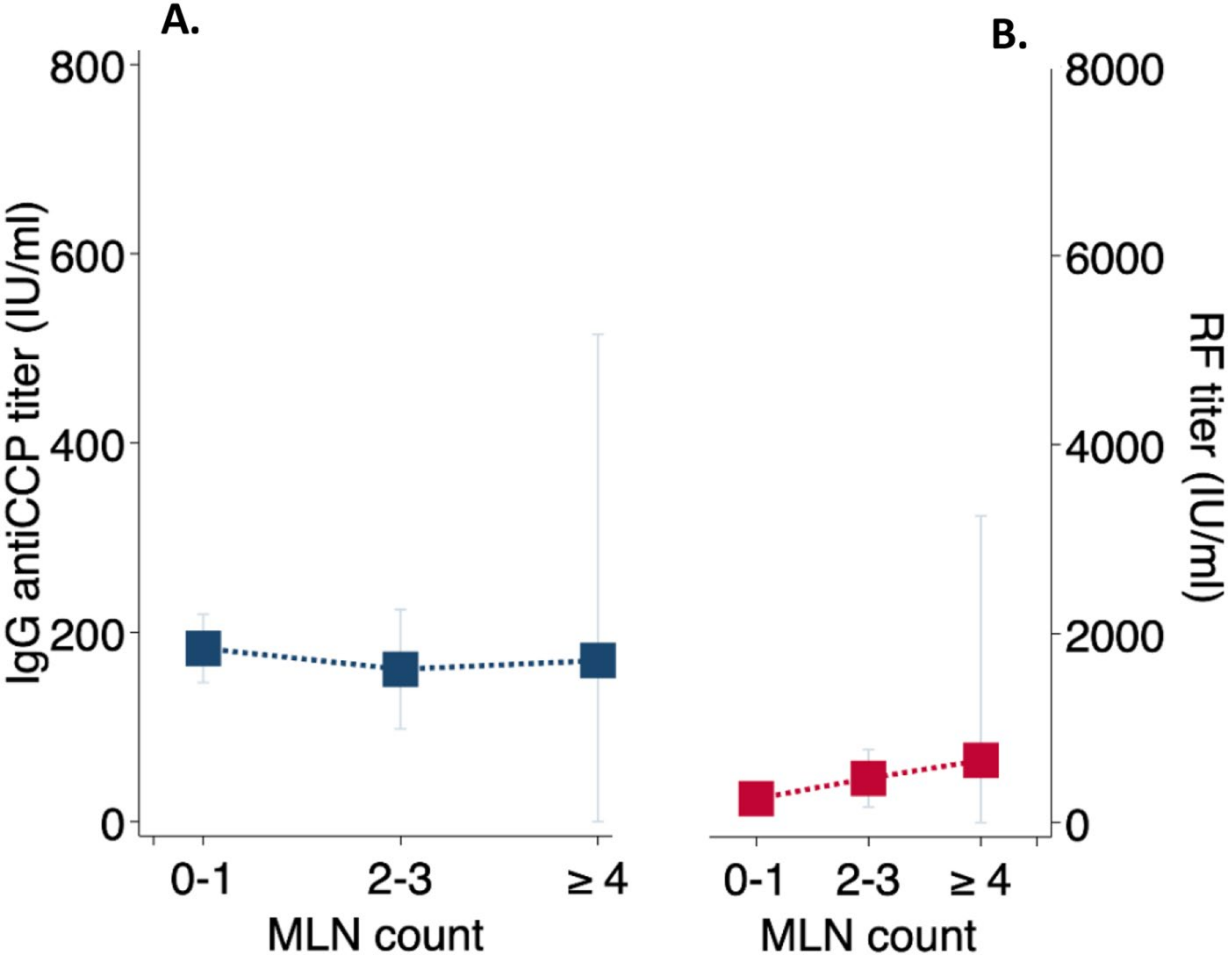
(**A**) IgG anti-CCP titer level remained constant in RA-ILD patients regardless of MLN count; (**B**) Increased MLN count correlated with proportional increase in RF titer in RA-ILD (y-axis truncated at 0 for ease of graphical representation); n = 58 for MLN count. *Abbreviations:* MLN = mediastinal lymphadenopathy, RF = rheumatoid factor, anti-CCP = anti-cyclic citrullinated peptide.

When comparing morphological CT features between groups, patients in the high-titer RF group had significantly higher rates of radiologic honeycombing (79% vs 50%; *P* = 0.008). There was no difference in rates of emphysema. Amongst patients with RA-ILD fifty-seven percent had MLN enlargement; most commonly located at the lower paratracheal region (station 4). In patients with MLN enlargement, the average lymph node size was 12.8 mm, and MLN size did not differ between high-titer and low-titer RF groups. MLN size was predicted by male gender (*P* = 0.001), and smoking history (*P* = 0.048) (Table 2). MLN enlargement was more common amongst patients with high-titer RF (*P* = 0.005). RF titer also positively correlated with MLN count, while anti-CCP titer did not (Fig. 1A–B).

**Table 2:** CT indices and mortality outcomes in RA-ILD.

Characteristics (n = 70)*	High-Titer RF Present (n = 42)	High-Titer RF Absent (n = 28)	*P*-value
**CT indices**			
CT Honeycombing, n (%)	33 (80)	14 (50)	0.008
Emphysema, n (%)	16 (43)	9 (33)	0.42
MLN ≥ 10 mm present, n (%)	24 (73)	9 (36)	0.005
MLN count, n (%)	1.5 (1.3)	0.8 (1.2)	0.07
**Mortality outcomes**			
Deceased or transplanted, n (%)	23 (55)	6 (21)	0.006
Mean survival time, months (± SD)	126 (96–156)	207 (172–242)	0.001
Crude mortality rate (events/100 person-yrs)	8.1 (5.4–12.1)	2.6 (1.2–5.7)	< 0.001
Unadjusted hazard ratio^^^ (95% CI)	3.0 (1.2–7.4)	–	0.016
Adjusted hazard ratio^^ƚ^ (95% CI)	2.8 (1.1–6.8)	–	0.028

*Exception: emphysema (n = 64); MLN presence/count (n = 58); Honeycombing (n = 69); Mean survival time = time to death or lung transplantation; ^Computed using Cox proportional hazard models; †Adjusted for composite GAP score (sex, age, forced vital capacity (FVC), diffusing capacity of the lungs for carbon monoxide (DLCO)).

### PFT and mortality outcomes in RA-ILD

**Figure 2:**
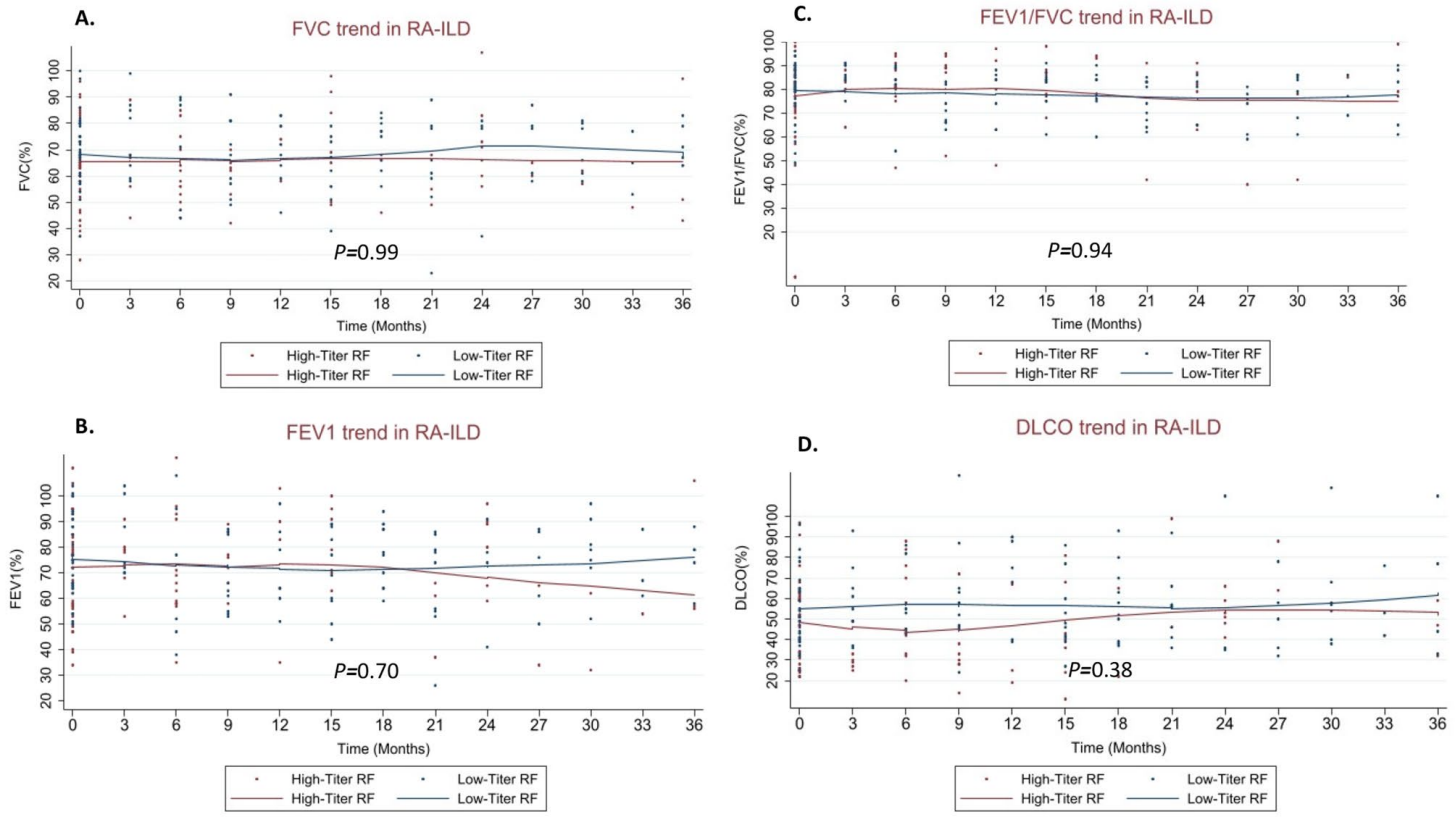
(**A**–**D**) Lung function decline as measured by percent predicted forced vital capacity (FVC) (**A**), percent predicted forced expiratory volume in 1 s (FEV_1_) (**B**), FEV_1_/FVC (**C**), and diffusing capacity of lung for carbon monoxide (DLCO) (**D**) over 36 months was not significantly different among high and low-titer RF groups. *P-values for mixed effect multi-level regression model assessing monthly change in lung function over time for the high-titer RF group.*

**Figure 3:**
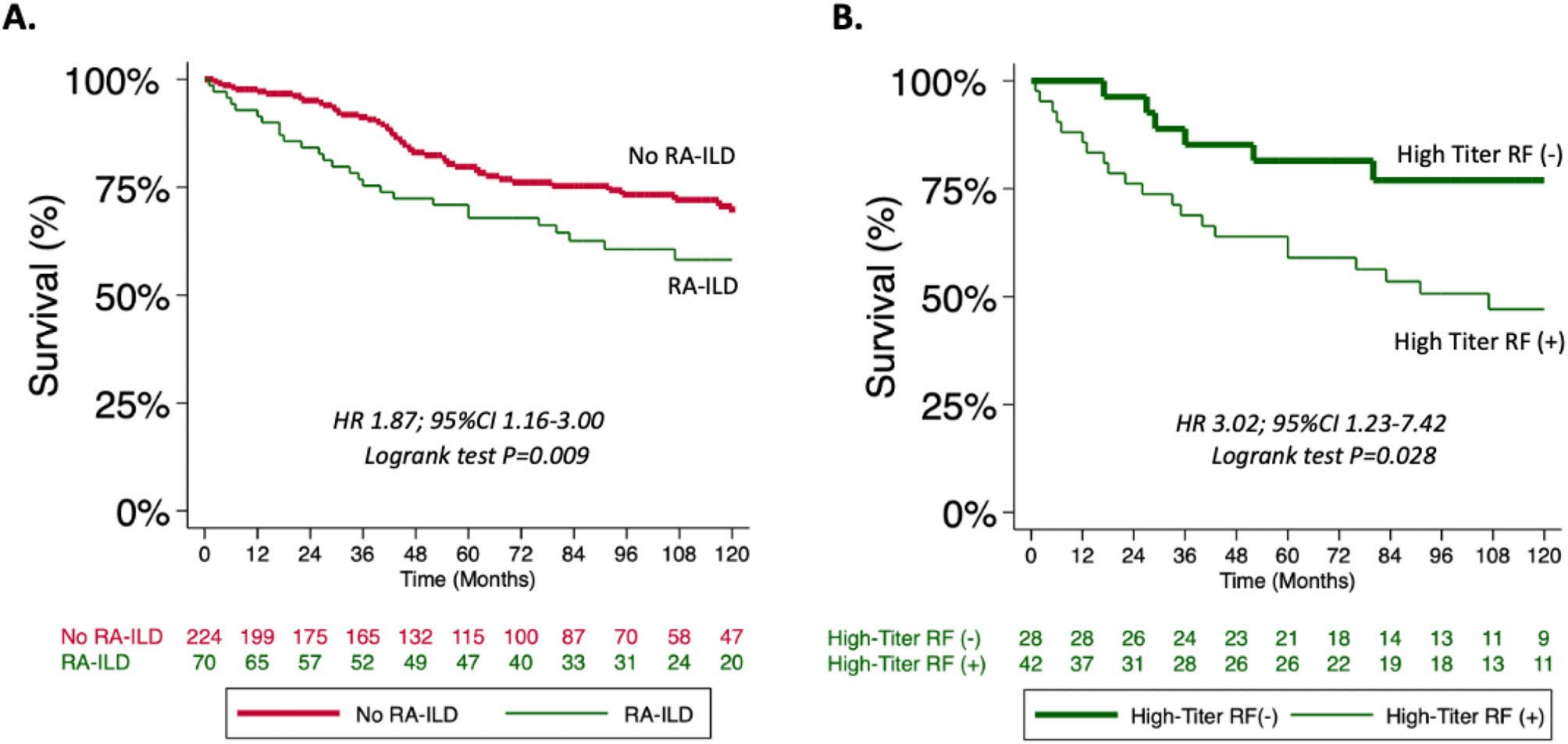
(**A**) Kaplan–Meier (KM) survival curves demonstrate transplant-free survival is significantly decreased in RA-ILD patients compared to other types of CTD-ILD; (**B**) Within the RA-ILD subgroup, KM survival curves demonstrate transplant-free survival is significantly decreased in the high-titer RF group (RF ≥ 60 IU/ml). Cox proportional hazard models with log-rank test were used for assessment of survival. *Abbreviations:* HR = hazard ratio, RF = rheumatoid factor, RA-ILD = rheumatoid arthritis related interstitial lung disease, CTD-ILD = connective tissue disease related interstitial lung disease.

When assessing unadjusted lung function changes on serial PFTs measured over 36 months, our study revealed no differences between the high-titer RF and low-titer RF groups (FVC + 0.05% vs − 4.89%; *P* = 0.99; FEV_1_ − 2.38% vs − 1.37%, *P* = 0.70; FEV_1_/FVC + 0.50% vs − 0.24%; *P* = 0.94; and DL_CO_ − 3.96% vs − 5.76%; *P* = 0.38) (Fig. 2). Mortality in the RA-ILD primary cohort was greater compared to other CTD-ILDs (HR 1.87; 95% CI 1.16–3.00; *P* = 0.009) (Fig. 3A). The presence of high-titer RF was also associated with a higher crude mortality rate (8.1 events/100 person-yrs vs 2.6 events/100 person-yrs; *P* < 0.001) and shorter mean survival time (126 months vs 207 months; *P* = 0.001) (Table 2). Mortality was increased amongst patients with high-titer RF in unadjusted analysis (HR 3.0; 95% CI 1.2–7.4; *P* = 0.016) and after adjustment for ILD severity using the GAP score (HR 2.8; 95% CI 1.1–6.8 *P* = 0.028) (Fig. 3B). Mortality outcomes did not differ when patients were stratified by presence or absence of high anti-CCP titers (E-Table 2).

## Discussion

This study is the first to examine the relationship between RF seropositivity, mediastinal lymphadenopathy, lung function decline, and transplant-free survival in RA-ILD patients. We found that patients with the highest RF titers (≥ 60 IU/ml) carry the greatest risk of disease progression and mortality. This increased hazard for death persists even after adjustment for the GAP score, a well-validated predictive index of disease severity across diverse forms of ILD including RA-ILD^22^. While age, male gender, smoking history, anti-CCP and RF seropositivity are well-known risk factors for the development of RA-ILD, a reliable biomarker for disease prognosis has yet to be identified for this patient population^23^. Recently, there has been increasing recognition that a common genetic background may exist between RA-ILD and IPF^24^. Despite this, investigations of well-established IPF-associated genetic mutations and serum biomarkers in RA-ILD patients has not led to a candidate prognostic marker.

Current knowledge regarding candidate biomarkers predicting mortality in RA-ILD has mostly been gleaned from the IPF literature. Recent studies evaluating prognostic biomarkers in ILD identified Krebs von den lungen-6 (KL-6) and surfactant protein-D (SP-D) as candidate serum biomarkers for IPF. However, sensitivity for these biomarkers in RA-ILD is low and routine testing is not universally available^25,26^. Human leukocyte antigen variants have also been associated with ILD susceptibility in RA patients, but not with prognosis^27^. In concordance with previous literature, anti-CCP titers were also significantly higher in our RA-ILD cohort but were not associated with increased mortality or lung function decline^23^. RF, an autoantibody secreted by specialized B-cells targeting the Fc region of IgG, was the first autoantibody described in RA. It has been postulated that RF directly contributes to the pathogenesis of RA by potentiating a cycle of immune complex formation and complement fixation, which leads to additional autoantibody production^28^. Nell et al. previously demonstrated an association between high-RF titer > 50 U/mL and progression of erosive joint disease in RA, further supporting its central role in pathogenesis and utility as a potential biomarker^29^. While RF seropositivity has been studied independently and in combination with anti-CCP seropositivity in the context of quantifying disease risk, our study is the first to demonstrate the association between this biomarker and mortality in RA-ILD^30^.

Genetic loci associated with the development of IPF have also recently been studied in RA-ILD. Specifically, it is postulated that RA-ILD pathogenesis may be driven by MUC5B overexpression which decreases airway ciliary clearance, resulting in parenchymal damage. This has been further supported by studies showing immunohistochemical staining co-locating with areas of MUC5B expression in areas of microscopic honeycombing in the lung^31^. While it is unknown if patients with high-titer RF seropositivity have MUC5B overexpression, our study identified a greater prevalence of CT honeycombing in the subgroup with high-titer RF seropositivity. Thus, improving our understanding of the genetic underpinnings of RA-ILD may contribute to the identification of additional biomarkers in the future.

Previously published work by our group has also demonstrated an association between MLN features on chest CT, decreased lung function, and increased mortality^14^. This relationship is most profound in IPF and is more common in patients with UIP pattern on chest CT, but has also been seen in patients with other CT patterns of pulmonary fibrosis. It has been postulated that MLN enlargement in IPF is caused by ongoing immunologic response in the lungs and the presence of a distinct phenotype of T-cells not seen in normal lung parenchyma^32,33^. The high frequency of UIP pattern, greater prevalence of MLN enlargement, and preponderance of tobacco use seen in both IPF and RA-ILD suggests shared pathobiology between both diseases and possibly similar outcomes. Tobacco use and smoking pack-years were more prevalent in our RA-ILD population compared to other CTD-ILDs, which is consistent with current evidence that cigarette smoking, a known risk factor for RA-ILD, may also trigger an immune response that results in epithelial cell injury of the lungs through the production of anti-CCP antibodies^23^. Our study lends credence to these findings, demonstrating that patients with high-titer RF in RA-ILD are more likely to have MLN enlargement, and elevated CCP titers in comparison to patients with low-titer RF. Furthermore, unlike anti-CCP titers, RF titer levels increase linearly with rising MLN count. Thus, the use of high-titer RF measurements in addition to MLN assessment may permit improved definition of disease prognosis in this population.

Interestingly, our study did not identify an association between RF seropositivity and lung function decline even though we found increased rates of CT honeycombing in the high-titer RF group. This was intriguing given that the UIP pattern on chest CT, which is most specifically characterized by honeycombing, is known to be associated with a poor prognosis in RA-ILD^34^. This might imply that other underlying immunologic or pathophysiologic mechanisms beyond lung function decline directly impact survival in patients with RA-ILD who have high-titer RF seropositivity.

This investigation had some limitations. It is possible that our inability to detect significant differences in lung function over time may have been due to our study sample size. Our follow-up time of 36 months may have been too short, although this seems less likely given that the mean survival of RA-ILD post-diagnosis is estimated at 3–5 years^35^. Further, our PFT results are comparable to those from others showing similar lung function decline over time in RA-ILD^36^. Our high-titer RF and low-titer RF groups may also represent heterogenous cohorts with respect to treatment with disease-modifying antirheumatic drugs (DMARDs), which could have impacted lung function and prognosis. Because we did not capture such data, we cannot comment on the influence of DMARDs in this analysis. Our findings are also limited by the single-center nature of this investigation. As our ILD clinic is located at a large tertiary academic referral center, it is possible that this study selected for more severe phenotypes of RA-ILD compared to the general population. However, baseline characteristics in our RA-ILD cohort were similar to those reported in other ILD literature. Additionally, as this study is a retrospective analysis it cannot elucidate the mechanisms underlying the association between elevated RF titer and MLN or mortality in RA-ILD. Thus, a larger multi-center prospective trial is needed to establish this relationship.

## Conclusion

In summary, high-titer RF seropositivity was associated with MLN enlargement, CT honeycombing, and decreased transplant-free survival in a retrospective cohort of patients with RA-ILD. Our findings suggest important clinical implications regarding the potential use of RF titer and MLN as independent prognostic markers of mortality in RA-ILD. Further studies are needed to elucidate the causal mechanisms of this relationship and identify factors predicting lung function decline in RA-ILD.

## Supplementary Information

Supplementary Information.
