# Supplementary material for: High-titer rheumatoid factor seropositivity predicts mediastinal lymphadenopathy and mortality in rheumatoid arthritis-related interstitial lung disease
Source: Sci Rep. 2021 Nov 24;11:22821. doi: 10.1038/s41598-021-02066-9 (PMC8613201; doi:10.1038/s41598-021-02066-9)
Supplement: Supplementary file 1 — Supplementary Information. [file 41598_2021_2066_MOESM1_ESM.pdf]

High-Titer Rheumatoid Factor Seropositivity Predicts Mediastinal Lymphadenopathy and Mortality in Rheumatoid Arthritis-related Interstitial Lung Disease

Albina Tyker, MD[1]; Iazsmin Bauer Ventura, MD, MS[2]; Cathryn T. Lee, MD[3]; Rachel Strykowski, MD[3]; Nicole Garcia, BS[3]; Robert Guzy, MD[3]; Renea Jablonski, MD[3]; Rekha Vij, MD[3]; Mary E. Strek, MD[3]; Jonathan H. Chung, MD[4]; Ayodeji Adegunsoye MD, MS[3]

1. Internal Medicine, University of Chicago, Chicago, IL, United States,
2. Rheumatology, University of Chicago, Chicago, IL, United States
3. Pulmonary/Critical Care, University of Chicago, Chicago, IL, United States
4. Radiology, University of Chicago, Chicago, IL, United States.

Corresponding Author:
Albina Tyker, MD
Department of Internal Medicine,
University of Chicago,
Chicago, IL, 60637
Albina.Tyker@uchospitals.edu

E-Table 1. Baseline Characteristics of CTD-ILD cohort.

Characteristics (n=294)*	RA-ILD (n=70)	Other CTD-ILD (n=224)	*P*-value
Age, mean (±SD)	62.8 (9.9)	56.7 (14.0)	< 0.001
Male gender, n (%)	27 (39)	60 (27)	0.06
Race/Ethnicity			
Caucasian, n (%)	39 (56)	106 (47)	0.22
African American, n (%)	21 (21)	49 (25)	0.38
Tobacco use, n (%)	45 (64)	72 (43)	0.003
Smoking, pk-yrs, mean (±SD)	23.0 (26.7)	7.7 (15.0)	< 0.001
FVC (% predicted) (±SD)	66.2 (15.1)	63.7 (20.0)	0.36
DL_{CO} (% predicted) (±SD)	52.0 (21.3)	56.0 (22.2)	0.22

**Exception: Tobacco use (n=237); FVC = forced vital capacity, (n=281); DLCO = diffusion lung capacity of carbon monoxide (n=255); CTD-ILD= connective tissue disease related interstitial lung disease; RA-ILD = rheumatoid arthritis related interstitial lung disease.*

E-Table 2. Mortality Outcomes in RA-ILD by anti-CCP Titer

**Characteristics (n=62)***	**High anti-CCP Titer Present (n=41)**	**High anti-CCP Titer Absent (n=21)**	***P*-value**
Mortality outcomes			
Deceased or transplanted, n (%)	17 (41)	9 (43)	0.92
Mean survival time, months (±SD)	152 (121-183)	150 (98-201)	0.94
Crude mortality rate (events/100 person-yrs)	5.6 (3.5-9.0)	6.5 (3.4-12.4)	0.77
Unadjusted hazard ratio^ (95% CI)	0.93 (0.41–2.09)	-	0.86
Adjusted hazard ratio^† (95% CI)	0.98 (0.43–2.21)	-	0.96

*High CCP titer = anti-CCP titer ≥ 100; *Exception: anti-CCP titers unavailable in 8 patients. Mean survival time = time to death or lung transplantation; ^Computed using Cox proportional hazard models; †Adjusted for composite GAP score (sex, age, forced vital capacity (FVC), diffusing capacity of the lungs for carbon monoxide (DLCO)).*
